# Microbiota That Affect Risk for Shigellosis in Children in Low-Income Countries

**DOI:** 10.3201/eid2101.140795

**Published:** 2015-02

**Authors:** Brianna Lindsay, Joe Oundo, M. Anowar Hossain, Martin Antonio, Boubou Tamboura, Alan W. Walker, Joseph N. Paulson, Julian Parkhill, Richard Omore, Abu S.G. Faruque, Suman Kumar Das, Usman N. Ikumapayi, Mitchell Adeyemi, Doh Sanogo, Debasish Saha, Samba Sow, Tamer H. Farag, Dilruba Nasrin, Shan Li, Sandra Panchalingam, Myron M. Levine, Karen Kotloff, Laurence S. Magder, Laura Hungerford, Halvor Sommerfelt, Mihai Pop, James P. Nataro, O. Colin Stine

**Affiliations:** University of Maryland School of Medicine, Baltimore, Maryland, USA (B. Lindsay, T.H. Farag, D. Nasrin, S. Li, S. Panchalingam, M.M. Levine, K. Kotloff, L.S. Magder, L. Hungerford, O.C. Stine);; Centers for Disease Control and Prevention/Kenya Medical Research Institute Research Station, Kisumu, Kenya (J. Oundo, R. Omore);; International Center for Diarrheal Disease Research, Mirzapur, Bangladesh (M.A. Hossain, A.S.G. Faruque, S.K. Das);; Medical Research Council, Basse, The Gambia (M. Antonio, U.N. Ikumapayi, M. Adeyemi, D. Saha);; Centre pour le Developpement des Vaccins du Mali, Bamako, Mali (B. Tamboura, D. Sanogo, S. Sow);; Wellcome Trust Sanger Institute, Hinxton, UK (A.W. Walker, J. Parkhill);; University of Maryland, College Park, Maryland, USA (J.N. Paulson, M. Pop);; University of Queensland, Brisbane, Queensland, Australia (S.K. Das);; University of Bergen, Bergen, Norway (H. Sommerfelt); Norwegian Institute of Public Health, Bergen (H. Sommerfelt);; University of Virginia School of Medicine, Charlottesville, Virginia, USA (J.P. Nataro)

**Keywords:** shigellosis, Shigella, bacteria, polymicrobial infection, Escherichia coli, enteroinvasive E. coli, EIEC, Lactobacillus, rotavirus, viruses, co-occurring pathogens, enteropathogens, microbiota, ipaH gene, diarrhea, children, low-income countries

## Abstract

Pathogens in the gastrointestinal tract exist within a vast population of microbes. We examined associations between pathogens and composition of gut microbiota as they relate to *Shigella* spp.*/e*nteroinvasive *Escherichia coli* infection. We analyzed 3,035 stool specimens (1,735 nondiarrheal and 1,300 moderate-to-severe diarrheal) from the Global Enteric Multicenter Study for 9 enteropathogens. Diarrheal specimens had a higher number of enteropathogens (diarrheal mean 1.4, nondiarrheal mean 0.95; p<0.0001). Rotavirus showed a negative association with *Shigella* spp. in cases of diarrhea (odds ratio 0.31, 95% CI 0.17–0.55) and had a large combined effect on moderate-to-severe diarrhea (odds ratio 29, 95% CI 3.8–220). In 4 *Lactobacillus* taxa identified by 16S rRNA gene sequencing, the association between pathogen and disease was decreased, which is consistent with the possibility that *Lactobacillus* spp. are protective against *Shigella* spp.–induced diarrhea. Bacterial diversity of gut microbiota was associated with diarrhea status, not high levels of the *Shigella* spp. *ipaH* gene.

## Abstract 2

Co-infection with *Shigella* spp. and other microbes modifies diarrhea risk.

Diarrheal disease contributes substantially to illness and death in children in low-income countries (*1*,*2*). Recent investigations of enteric illness have shown many cases with >1 pathogen identified (*3*–*5*). The paradigm of 1 pathogen and 1 disease has been questioned with the advent of microbiological and molecular detection methods that have lower limits of detection. Children in developing countries are exposed to an array of pathogenic organisms. Recent studies have shown a complex relationship between gut microbiota and diarrheal illness; children with severe illness tend to have a less diverse microbiota and a predominance of specific genera of organisms (*6*). Molecular-based approaches to pathogen detection enhance the ability to quantify the abundance of pathogens shed in the stool.

Two recent studies of children in low-income countries have highlighted the need for pathogen quantitation. Lindsay et al., using the Global Enteric Multicenter Study (GEMS) specimen collection, found that 80% of controls and 89% of case-patients had detectable levels of *Shigella* spp. (*7*). To identify which children had shigellosis, Lindsay et al. determined a quantitative threshold and, when applied, it identified twice as many cases compared with standard culture. Platts-Mills et al., in a study of populations with a high prevalence of malnutrition and enteric infections in Tanzania, compared samples taken before and during diarrheal episodes (*8*). They did not find an association between the presence of any pathogen and diarrhea for 15 pathogens studied (rotavirus, adenovirus, astrovirus, norovirus, sapovirus, *Cryptosporidium* spp., *Giardia lamblia*, *Campylobacter jejuni*, *Clostridium difficile*, *Salmonella* spp., Shiga-toxigenic *Escherichia coli*, *Shigella* spp./enteroinvasive *E. coli* [EIEC], enterotoxigenic *E. coli* [ETEC], typical enteropathogenic *E. coli* [tEPEC], and enteroaggregative *E. coli* [EAEC]). However, when they considered quantity of pathogen on a continuous scale, 3 organisms (rotavirus, astrovirus, and *Shigella* spp.) were associated with diarrhea.

In disease-endemic settings, detection of multiple enteropathogens in asymptomatic and symptomatic children is common (*4*,*9*). Samples from one third of patients with diarrhea in a hospital study in Kolkata, India, contained >1 pathogen. Negative associations were demonstrated between *Shigella* spp. and rotavirus and *Shigella* spp. and *Vibrio cholerae* (*3*). However, a limitation of this study was that it was conducted only in patients with diarrhea. Thus, differential comparisons could not be made between pathogen associations in diarrheal and nondiarrheal samples. A recent study by Taniuchi et al. reported the etiology of diarrheal episodes by using molecular methods in Bangladeshi children during their first year of life. They found that multiple enteropathogens were present by the first month of life in stool specimens from healthy children and from children with diarrhea (*4*). If multiple pathogens are present, they might interact to increase or decrease the probability of symptomatic infection. Bhavnani et al. reported synergistic effects in rotavirus–*G. lamblia* and rotavirus–*E. coli* infections, in which the presence of these co-occurring organisms increased the probability of disease (*10*).

The gut microbiota are composed of thousands of species that might play a role in the risk for diarrhea. Some *Lactobacillus* and *Veillonella* species are potentially protective against diarrhea or serve as markers of healthy gut microbiota (*11*–*13*) Probiotic activity has been associated with some *Lactobacillus* spp., bifidobacteria, *Veillonella* spp., *Streptococcus* spp., *Enterococcus* spp., nonpathogenic *E. coli*, and *Saccharomyces boulardii* (*13*). Randomized clinical trials have investigated the role of some *Lactobacillus* spp. in treating infectious diarrhea and identified that these organisms can provide a benefit in the treatment of acute, infectious, watery diarrhea in infants and young children (*12*). In this study, we examined relationships between *Shigella* spp.*/*EIEC, microbiota, and diarrhea by using 16S rRNA marker gene surveys of stool specimens from a large international study of diarrhea in children <5 years of age (*9*).

## Methods

### Study Design and Participants

We used stool specimens collected from children <5 years of age who participated in a matched case–control study of moderate-to-severe diarrhea sponsored by the GEMS consortium (*14*,*15*). In brief, the GEMS was a prospective case–control study of infants and young children at 7 sites in sub-Saharan Africa and southern Asia. Case-patients with moderate-to-severe diarrhea were enrolled when they came to a health clinic. Moderate-to-severe diarrhea eligibility criteria included dehydration (sunken eyes, loss of normal skin turgor, or a decision to initiate intravenous hydration), the presence of blood in the stool (dysentery), or a clinical decision to hospitalize the child. In the GEMS, matching controls (for sex, age, and community) were sampled from a demographic surveillance database of the area and included if they reported no diarrhea within the previous 7 days. For this study, 4 of the 7 GEMS sites (The Gambia, Mali, Kenya, and Bangladesh) elected to participate in further molecular characterization of their samples. When samples were sent for analysis, it was not required that their matched specimen be included, which resulted in disruption of matches. Including only samples with the complete matched set would have limited our sample size. Therefore, we included age and location as covariates in our analysis but did not analyze samples as matched sets.

All specimens for this study were collected during December 2007–December 2009. One specimen was collected for each child at the time of enrollment. The Institutional Review Boards at all participating institutions reviewed and approved the protocol.

### Specimen Collection

Stool specimens were handled according to the GEMS protocol (*16*). DNA was isolated from frozen stool specimens by using a bead beater with 3-mm diameter solid glass beads (Sigma-Aldrich, St. Louis, MO, USA) and, subsequently, with 0.1-mm zirconium beads (BioSpec Products, Inc., Bartlesville, OK, USA) to disrupt cells. The cell slurry was then centrifuged at 16,000 × *g* for 1 min, and the supernatant was processed by using QIAamp DNA Stool Extraction Kit (QIAGEN, Valencia, CA, USA). Extracted DNA was precipitated with ethanol and shipped to the United States.

### Detection of EAEC, tEPEC, ETEC, Rotavirus, Norovirus, *G. lamblia*, and *Cryptosporidium* spp.

Diagnostic microbiological methods for rotavirus, norovirus, *G. lamblia*, *Cryptosporidium* spp., and diarrheagenic *E. coli* (EAEC, tEPEC, and ETEC) were conducted at each site as described by Panchalingham et al. (*16*). In brief, *E. coli* isolates were selected from MacConkey agar plates and tested by using motility indole ornithine medium. EAEC, tEPEC, and ETEC pathotypes were identified by using PCRs for known virulence determinants of each pathogen (ETEC: heat-labile and heat-stable enterotoxins; tEPEC: intimin [*eae*] and bundle-forming pilus; and EAEC: *aatA* and *aaiC* genes).

Rotavirus was detected by using the ProSpecT ELISA Rotavirus Kit (Oxoid, Basingstoke, UK). Norovirus genogroups GI and GII were detected by using a multiplex PCR specific for synthesized complementary DNA after viral RNA extraction and reverse transcription. *G. lamblia* and *Cryptosporidium* spp. were detected by using commercially available immunoassays (TechLab, Blacksburg, VA, USA) following the manufacturer’s protocols. Although the GEMS study tested for other enteric pathogens, our analysis included only pathogens that were present in ≥10 specimens. This lower limit was chosen after examination of the distribution of positive samples for each pathogen and to avoid statistical testing with small sample sizes.

### Quantitative PCR for Detection of *ipaH* Gene in *Shigella* spp. and EIEC

Each stool DNA specimen was tested by using a quantitative PCR (qPCR) for *Shigella*/EIEC that included the 7500/700 Fast Real-Time PCR System, software V2·0·5, and SYBR green–based fluorescent dye (Applied Biosystems, Foster City, CA, USA). Details on primer design, PCR conditions, and standard curve analysis have been reported elsewhere (*4*,*7*,*17*). *Shigella*/EIEC was identified by using primers specific for the *ipaH* gene (*7*). We used a cutoff of 14,000 *ipaH* gene copies to distinguish children shedding low levels of *Shigella* spp. from those shedding high levels of *Shigella* spp. in their stools. The threshold was established by constructing receiver-operating characteristic curves to determine sensitivity and specificity of incremental increases in levels of *ipaH* compared with disease status and set on the basis of the point that maximized sensitivity and specificity (*7*). Stool specimens with high and low levels of *ipaH* indicate the relative amount of *Shigella* spp. detected.

### 16S rRNA Gene Sequencing and Analysis and Identification of *C. jejuni*

DNA was amplified by using universal primers specific for the V1–V3 region of the 16S rRNA gene in bacteria (518R [5′-CAATTACCGCGGCTGCTGG-3′] and 27F [5′-AGAGTTTGATCCTGGCTCAG-3′]). Individual reads were filtered for quality by using custom in-house scripts that removed low-quality sequences as described (*6*). Remaining high-quality sequences were separated into sample-specific sets according to barcodes. Conservative operational taxonomic units (OTUs) were clustered by using DNACLUST with parameters (–r 1 and 99% identity clusters) to ensure that the definition of an OTU was consistent across all samples (*18*). For taxonomic identification, a representative sequence from each OTU was aligned to the Ribosomal Database (RDP) (rdp.cme.msu.edu, release 10.4) by using BLASTn (http://blast.ncbi.nlm.nih.gov/Blast.cgi) with long word length (–W 100) to detect only nearly identical sequences (*19*). Sequences without a nearly identical match to the RDP (>100-bp perfect match and >97% identity, as defined by BLAST) were marked as being unassigned and assigned a unique OTU identifier. If a sample contained taxa classified as *C. jejuni,* we identified this sample as positive for *C. jejuni*.

### Statistical Analysis

Associations between high levels of *ipaH* and each additional pathogen, stratified by diarrheal status, were assessed for children with moderate or severe diarrhea and for controls by using separate logistic regression models with high levels of *ipaH* as the dependent variable. Logistic regression with moderate-to-severe diarrhea as the outcome of interest was then conducted to test for the interaction between high levels of *ipaH* and either 1) pathogenic microorganisms tested for by the GEMS or 2) species identified by 16S rRNA gene sequencing. All models were adjusted for potential confounding caused by location and age with categorical location and age terms in the model.

Statistical modeling was used to examine whether microbes interact to effect diarrhea risk. To assess whether the risk caused by having *Shigella* spp. and an additional microbe differed from the product of the risks caused by having each microbe separately (i.e., multiplicative interaction), we used a logistic regression model with an interaction term containing the level of *ipaH* and the additional microbe of interest (Technical Appendix Figure). To assess whether the excess risk of having *ipaH* and an additional microbe differed from the sum of the excess risk of having each separately (i.e., additive interaction), we estimated the relative excess risk caused by the interaction (RERI, also called the interaction contrast ratio [ICC]) (*20*–*22*). An RERI = 0 indicates no additive interaction, an RERI>0 suggests positive additive interaction, and an RERI<0 suggests negative additive interaction. Ninety-five percent CIs were estimated by using the Hosmer-Lemeshow procedure (*23*,*24*). All statistical analysis was performed by using SAS version 9·2 (SAS Institute, Cary, NC, USA) and R 2.15 (*25*), and p values <0.05 were considered significant.

## Results

A total of 3,035 (1,735 nondiarrheal and 1,300 diarrheal) stool specimens from children <5 years of age from The Gambia, Mali, Kenya, and Bangladesh were examined for *Shigella* spp. by identification of *ipaH* by qPCR; for *G. lamblia*, *Cryptosporidium* spp., and rotavirus by ELISA; for norovirus by PCR; for EAEC, ETEC, or tEPEC by culture and subsequent PCR; and for *C. jejuni* by 16S rRNA gene sequencing. Characteristics of samples are shown in Table 1. Diarrheal samples had a significantly higher number of pathogens (diarrheal mean 1.4, nondiarrheal mean = 0.95; p<0.05).

**Table 1:** Characteristics and pathogen abundance in children with cases of moderate-to-severe diarrhea and controls in low-income countries*

Characteristic	Cases, n = 1,300	Controls, n = 1,735	p value	Total, n = 3,035
Male sex	727 (56)	965 (56)	0.8677	1,692 (56)
Age, mo, mean (SD)	16.7 (12)	17.8 (12)	0.0073	17.39 (12)
0–5	156 (12)	177 (10)	0.1167	333 (11)
6–11	413 (32)	465 (27)	0.0028	878 (29)
12–23	431 (33)	617 (35)	0.1674	1,048 (35)
24–35	185 (14)	311 (18)	0.0064	496 (16)
36–59	115 (9)	165 (10)	0.5318	280 (9)
Country	NA	NA	NA	NA
The Gambia	356 (27)	408 (23)	0.0151	764 (25)
Mali	103 (8)	114 (7)	0.1524	217 (7)
Kenya	636 (49)	779 (45)	0.0279	1,415 (47)
Bangladesh	205 (16)	434 (25)	<0.0001	639 (21)
*ipaH* gene copies ≥14,000	277 (22)	127 (7)	<0.0001	404 (13)
Rotavirus	183 (14)	41 (2)	<0.0001	224 (7)
Norovirus genogroups GI or GII	127 (10)	141 (8)	0.1146	268 (9)
*Giardia lamblia*	230 (18)	373 (22)	0.0093	603 (20)
*Cryptosporidium *spp.	142 (11)	87 (5)	<0.0001	229 (7)
tEPEC	106 (8)	104 (6)	0.0203	210 (7)
EAEC	253 (19)	347 (20)	0.7124	600 (20)
ETEC	181 (14)	148 (8)	<0.0001	329 (11)
*Camplylobacter jejuni*	341 (26)	264 (15)	<0.0001	605 (20)
Total pathogens, mean (SD)	1.4 (0.9)	0.9 (0.9)	<0.0001	1.2 (0.9)

*Values are no. (%) children unless otherwise indicated. NA, not applicable; tEPEC, typical enteropathogenic *Escherichia coli*; EAEC, enteroaggregative *E. coli*; ETEC, enterotoxigenic *E. coli*.

### Associations and Interaction Effects of Co-occurring Pathogens

**Figure 1:**
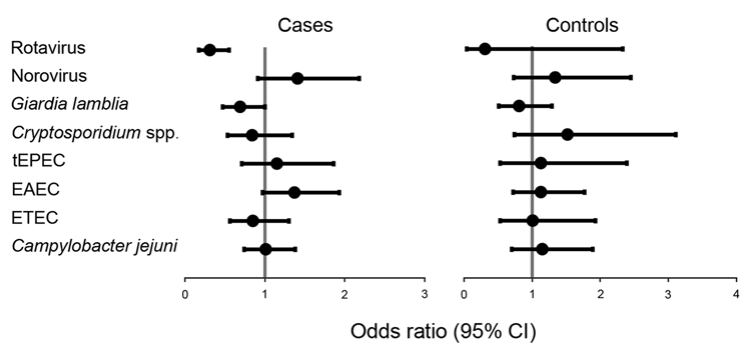
Association of co-occurring pathogens with high levels of *ipaH* gene of *Shigella* spp. in stool specimens of children with diarrhea (cases) and children without diarrhea (controls) in low-income countries. Dark circles indicate means, and error bars indicate 95% CIs. tEPEC, typical enteropathogenic *Escherichia coli*; EAEC, enteroaggregative *E. coli*; ETEC, enterotoxigenic *E. coli*.

In 69% (278/404) of samples with high levels of *ipaH* (71% of diarrheal samples with high levels of *ipaH* and 64% nondiarrheal samples with high levels of *ipaH*), we identified an additional pathogen. In samples with low levels of *ipaH*, we identified an additional pathogen in 69% (1,815/2,631; 80% of diarrheal samples with low levels of *ipaH* and 62% of nondiarrheal samples with low levels of *ipaH*). After adjusting for age and location, rotavirus exhibited a negative association with high levels of *ipaH* in case-patients (odds ratio [OR] 0.31, 95% CI 0.17–0.55). In case-patients and controls, no other pathogen showed an association (Figure 1; Technical Appendix Table 1).

Of the 8 pathogens tested, none showed any interaction effects. However, rotavirus was negatively associated with high levels of *ipaH* (Table 2), and only 15 samples that had high levels of *ipaH* were positive for rotavirus; 14 of these 15 samples were diarrheal samples. The presence of rotavirus and high levels of *ipaH* resulted in an OR of 29 (95% CI 3.8–220) for diarrheal risk. Although this point estimate was much higher than the expected additive effect of 11, this result and results of tests for interaction were not statistically significant (Table 3).

**Table 2:** Interaction between level of *Shigella* spp.*/EIEC ipaH* gene and rotavirus and association with moderate-to-severe diarrhea in children in low-income countries*

Rotavirus status†	Low level of *ipaH* gene			High level of *ipaH *gene		OR (95% CI) for *ipaH* gene within strata of rotavirus status
	No. with MSD/ no. without MSD	OR (95% CI)		No. with MSD/ no. without MSD	OR (95% CI)	
Negative	854/1,568	1.00 (reference)		263/126	4.01 (3.18–5.05)	4.01 (3.18–5.05)
Positive	169/40	7.56 (5.29–10.80)		14/1	28.85 (3.77–220)	3.82 (0.48–30.00)
OR (95% CI) for rotavirus within strata of *ipaH* gene status	NA	7.55 (5.29–10.80)		NA	7.20 (0.93–55.57)	Expected additive = 10.57

*EIEC, enteroinvasive *Escherichia coli*; MSD, moderate-to-severe diarrhea; OR, odds ratio; NA, not applicable. †Model is unconditional and adjusted for age and location.

**Table 3:** Measures of additive and multiplicative interactions of co-occurrence of *Shigella* spp. and another pathogen or *Lactobacillus* spp. in children in low-income countries*

Pathogen or organism	OR_10_† (95% CI)	OR_01_‡ (95% CI)	OR_11_§ (95% CI)	RERI (95% CI)	Additive p value	Multiplicative p value
*C. jejuni*	3.84 (2.98–4.95)	1.97 (1.62–2.40)	5.55 (3.67–9.15)	0.79 (−2.15 to 3.73)	0.62	0.29
ETEC	3.77 (2.97–4.80)	1.69 (1.32–2.18)	4.65 (2.32–9.30)	0.15 (−3.20 to 3.52)	0.92	0.41
tEPEC	3.69 (2.91–4.69)	1.36 (1.02–1.83)	4.09 (2.01–8.33)	0.03 (−2.99 to 3.06)	0.99	0.61
*Cryptosporidium* spp.	3.79 (2.99–4.81)	2.40 (1.78–3.24)	4.95 (2.28–10.75)	−0.27 (−4.27 to 3.73)	0.91	0.16
Rotavirus	4.01 (3.18–5.05)	7.56 (5.29–10.80)	28.85 (3.77–220)	18.29 (−40.32 to 76.91)	0.54	0.96
Norovirus genogroups GI or GII	3.65 (2.87–4.64)	1.14 (0.86–1.51)	4.00 (2.08–7.71)	0.21 (−2.56 to.98)	0.88	0.91
EAEC	3.59 (2.78–4.63)	0.87 (0.71–1.07)	3.29 (2.06–5.26)	−0.17 (−1.92 to 1.58)	0.85	0.86
*Giardia lamblia*	3.65 (2.84–4.69)	0.83 (0.68–1.02)	2.70 (1.63–4.49)	−0.78 (−2.83 to 0.83)	0.35	0.70
*Lactobacillus* taxon						
KLDS 1.0718	**4.10 (3.19–5.26)**	**1.25 (1.01–1.55)**	**2.42 (1.41–4.13)**	**−1.93 (−3.56 to 0.29)**	**0.02**	**0.02**
* L. salivarius*	4.12 (3.20–5.31)	1.77 (1.45–2.16)	3.88 (2.35–6.38)	−1.02 (−3.18 to 1.14)	0.36	**0.03**
* L. ruminis*	**4.30 (3.19–5.79)**	**0.82 (0.69–0.96)**	**2.20 (1.55–3.13)**	**−1.92 (−3.36 to 0.47)**	**0.01**	**0.05**
DJF RP24	**4.59 (3.41–6.16)**	**0.85 (0.72–0.99)**	**1.99 (1.39–2.85)**	**−2.44 (−3.93 to 0.95)**	**0.001**	**0.01**
TSK G32–2	**3.89 (3.08–4.92)**	**1.40 (1.07–1.85)**	**1.60 (0.59–4.30)**	**−2.69 (−4.55 to 0.84)**	**0.004**	**0.02**

*OR, odds ratio; RERI, relative excess risk caused by the interaction; ETEC, enterotoxigenic *Escherichia coli*; tEPEC, typical enteropathogenic *E. coli*; EAEC, enteroaggregative *E. coli*. Boldface indicates significance (p≤0.05). Under an additive interaction, OR_11_ – OR_10_ – OR_01_ + 1 = 0. Under a multiplicative interaction, OR_11_ = OR_10_OR_01_. †OR caused by having a high level of the *ipaH* gene alone (in the absence of the other pathogen or *Lactobacillus* taxon). ‡OR caused by having the other pathogen or *Lactobacillus* taxon alone (in the absence of a high level of the *ipaH *gene). §OR caused by having a high level of the *ipaH* gene and another pathogen or *Lactobacillus* taxon (relative to having neither).

### Interaction Effects of *Lactobacillus* and *Veillonella* Taxa

**Figure 2:**
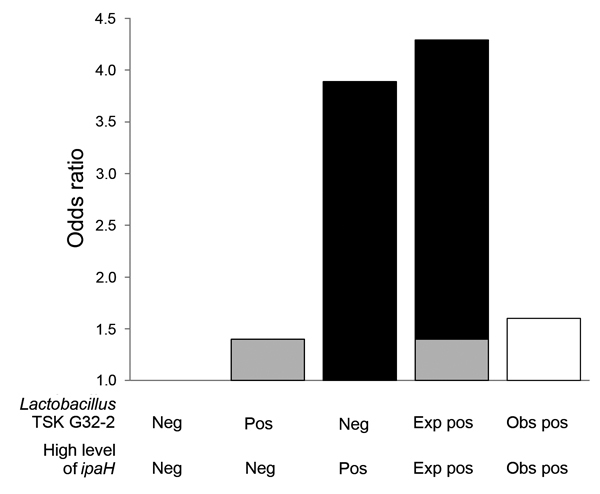
Departure from additivity between level of *ipaH* gene and presence of *Lactobacillus* taxon TSK G32-2 on odds of moderate-to-severe diarrhea in children in low-income countries. The reference group is TSK G32-2 negative, low level of *ipaH*. The observed combined joint effect of a high level of the *ipaH* gene and TSK G32-2 was lower than the expected additive effect. Gray bars indicate effect of TSK G32-2; black bars indicate effect of high levels of *ipaH*; and white bar indicates observed joint effect of TSK G32-2 and high levels of *ipaH*. Neg, negative; Pos, positive; Exp, expected; Obs, observed.

A total of 61 *Lactobacillus* and *Veillonella* taxa were identified by 16S rRNA gene sequencing. Analyses were limited to 31 taxa that co-occurred in samples with high levels of *ipaH* ≥10 times. We tested for interaction between high levels of *ipaH* and the identified 16S rRNA gene taxon by using a logistic regression model adjusted for age and location, including an interaction term between level of *ipaH* and taxon presence (Technical Appendix Table 2). Five taxa showed significant negative multiplicative interactions identified by an interaction term with p ≤ 0·05. Of these taxa, *Lactobacillus ruminis* (RERI −1.92, 95% CI −3.36 to 0.47), *Lactobacillus* DJF-RP24 (RERI −2.44, [95% CI −3.93 to 0.95), *Lactobacillus* KLDS 1.0718 (RERI −1.93, 95% CI −3.56 to 0.29), and *Lactobacillus* TSK G32.2 (RERI −2.69, 95% CI −4.55 to 0.84]) showed additive interactions (Table 3). The combined effect of high levels of *ipaH* in the presence of *L. ruminis, Lactobacillus* DJF RP24, *Lactobacillus* KLDS 1.0718, or *Lactobacillus* TSK G32.2 was lower than expected (Figure 2; Technical Appendix Table 3), which suggested an antagonistic interaction or a decreased association with diarrhea when specific *Lactobacillus* taxa were present than versus when they were absent. When we tested the 4 *Lactobacillus* taxa against 8 additional pathogens; no additive interaction was observed (Technical Appendix Table 4).

### 16S rRNA Gene–based Bacterial Community Profiles

**Figure 3:**
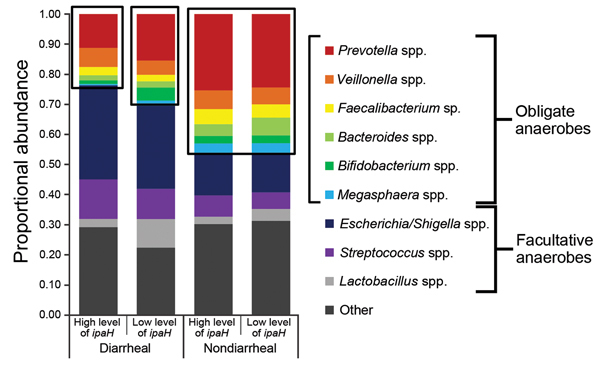
Overall 16S rRNA gene–based bacterial community profiles (proportional abundance) of diarrheal samples with high levels of *ipaH* gene (n = 277), diarrheal samples with low levels of *ipaH* gene (n = 1,023), nondiarrheal samples with high levels of *ipaH* gene (n = 127), and nondiarrheal samples with low levels of *ipaH* gene (n = 1,608) from children in low-income countries. Other indicates sequences that were not identified as 1 of the 9 most abundant taxa or did not have good (>100 bp exact match, >97% identity) matches with isolate sequences from the Ribosomal Database Project (*19*).

The proportional abundance of the 9 most common genera identified by 16S rRNA marker gene sequencing differed among stool specimens with high and low levels of *ipaH* and among specimens from children with and without diarrhea (Figure 3). Overall composition of diarrheal stool specimens had an increased relative proportional abundance of facultative anaerobes when compared with composition of nondiarrheal stool specimens, even when diarrheal stool specimens had low levels of *ipaH* (mean diarrheal specimens 0.47, mean nondiarrheal specimens 0.22; p<0.0001). Overall, diarrheal stool specimens with high levels of *ipaH* had the lowest proportion abundance of *Prevotella* spp. (0.11), diarrheal stool samples with low levels of *ipaH* had the second lowest proportional abundance (0.15), and nondiarrheal stool specimens with high and low levels of *ipaH* had similar proportion abundances for *Prevotella* spp. (0.25 and 0.24, respectively; p = 0.63).

*Shigella* spp. identified by the *ipaH* qPCR were a subset of the genera *Escherichia/Shigella* identified by 16S rRNA gene sequencing, but could not be distinguished from commensal strains. Diarrheal specimens had a larger proportion of members of the genera *Escherichia/Shigella* (p<0.0001) than nondiarrheal specimens, regardless of levels of *ipaH*. However, diarrheal stool specimens with high levels of *ipaH* had a significantly higher proportion of *Escherichia/Shigella* sequences (31%; p<0.0001) than did diarrheal stool specimens with low levels of *ipaH*.

**Figure 4:**
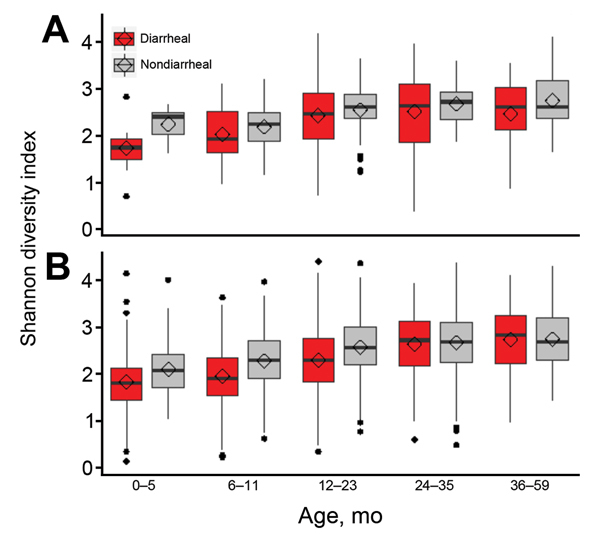
Shannon diversity index for diarrheal and nondiarrheal samples with high (A) and low (B) levels of *Shigella* spp. *ipaH* gene stratified by age group for children in low-income countries. Box and whisker plot indicates distribution of diversity index for each group. The upper whisker extends from the 75th percentile to the highest value that is ≤1.5× the interquartile range (IQR) of the hinge (upper end) or the distance between the first and third quartiles. The lower whisker extends from the hinge (lower end) to the lowest values 1.5× the IQR of the hinge. Diamonds indicate means and horizontal lines indicate medians. Points outside the ends of the whiskers are outliers beyond 1.5× the IQR of the hinge.

Members of the genus *Veillonella* was found equally in diarrheal and nondiarrheal stool specimens. Streptococci were found more often in diarrheal stool specimens, regardless of levels of *ipaH*. There was no association between Shannon diversity indices and levels of *ipaH* (p = 0.95), although diversity was significantly associated with age, location, and moderate-to-severe diarrhea (p<0.0001, p = 0.004, and p<0.0001, respectively) (Figure 4).

## Discussion

In this study, we used qPCR and 16S rRNA gene sequencing to identify interactions between *Shigella*/EIEC and co-occurring enteric pathogens or microbes within the gut microbiota in children with moderate-to-severe diarrhea. We explored these interactions in stool specimens obtained from children with and without diarrhea who participated in the international GEMS study. We used detection of the *ipaH* gene as an indicator of *Shigella*/EIEC infection because this molecular method is highly sensitive and specific (*4*,*26*,*27*). Use of quantitative identification methods, rather than colonization (*7*,*8*), is advantageous for identifying true disease associations. We found that 69% (1,815/2,631) of stool samples with high levels of *ipaH* had a co-occurring pathogen.

This study confirms the findings of the negative association between rotavirus and *Shigella* spp. in polymicrobial infections in diarrheal patients in India and The Gambia (*Shigella* spp./rotavirus: OR 0.36, 95% CI 0.14–0.92) (*3*,*28*). Although we identified correlations between rotavirus and *Shigella* spp. as determined by levels of *ipaH*, no pathogen showed an antagonistic or synergistic interaction on the odds of moderate-to-severe diarrheal illness. Although all but 1 of the rotavirus-positive samples that had high levels of *ipaH* were associated with diarrhea cases, the negative association between these pathogens gave us limited sample size to assess a possible synergistic effect. A synergistic effect between high levels of *ipaH* and rotavirus (RERI/ICC 9.9, 95% CI 2.6–28.4) was previously observed in Ecuador, and it was concluded that pathogenic potential appears to be enhanced during co-infection (*10*). Our RERI/ICC was 18, but the effect was not significant, possibly because of small sample size.

The GEMS was designed as a matched case–control study of thousands of stool samples from persons matched by age, sex, community, and time. A possible limitation of our methods is that we did not use matched pairs but rather adjusted for country-level location and age by using statistical modeling. This method was used because of the large number of broken matched pairs. Thus, inclusion of all samples greatly increased our sample size. Generally, the lack of an appropriate matched analysis biases the effect on estimates toward the null. However, studies have shown that including samples with missing data and adjusting by using confounding terms is valuable, particularly with a larger sample size (*29*,*30*). We adjusted for location at the country level, and the GEMS matched case-patients at the community level, which could fail to adequately address bias, particularly when one considers that the distribution of case-patients and controls differed at multiple sites and that *Shigella* spp. are more common in Bangladesh. An additional limitation of our study design is that, given a cross-sectional study sample set, we were unable to identify temporal associations and to attribute cause and effect. Furthermore, associations between pathogens and taxa may be explained by seasonality, and further work should be conducted to investigate this as a possible explanation. Finally, the 16S rRNA gene-sequencing method used to identify taxa of interest is limited because our identification was only as precise as the RDP attributed taxonomy. Thus, further characterization and species-specific identification are warranted in uncultured bacteria such as *Lactobacillus* KLDS 1.0718.

A previous study showed cross-sectional differences in stool microbiota of children with diarrhea compared with children without diarrhea in low-income countries (*6*). Our study showed that the composition of microbiota is more closely associated with diarrheal status than with co-infection by *Shigella* spp./EIEC as measured by high levels of *ipaH*. Microbiota in stools of children without diarrhea but who had high levels of *ipaH* (i.e., were colonized with *Shigella* spp./EIEC) were more similar to microbiota of healthy children with low levels of *ipaH* than microbiota in samples from children with diarrhea and high levels of *ipaH*.

In low-income countries, infection/colonization with pathogens occurs commonly in persons without diarrhea. Our study found little evidence of interaction between *Shigella* spp. and co-occurring pathogens. Although the cross-sectional study design precludes strong statements of cause and effect, our data are consistent with the possibility that some *Lactobacillus* taxa naturally occurring in the gut and are protective against *Shigella* spp.–induced diarrhea. Future studies should continue to consider the effects of co-occurring species.

**Technical Appendix.** Supplementary information regarding microbiota tested for their effects on risk for shigellosis in children in low-income countries.

## References

[1] Lozano R, Naghavi M, Foreman K, Lim S, Shibuya K, Aboyans V, Global and regional mortality from 235 causes of death for 20 age groups in 1990 and 2010: a systematic analysis for the Global Burden of Disease Study 2010. Lancet. 2012;380:2095–128. 10.1016/S0140-6736(12)61728-0 23245604 PMC10790329

[2] Walker CL, Aryee MJ, Boschi-Pinto C, Black RE. Estimating diarrhea mortality among young children in low and middle income countries. PLoS ONE. 2012;7:e29151. 10.1371/journal.pone.0029151 22235266 PMC3250411

[3] Lindsay B, Ramamurthy T, Sen Gupta S, Takeda Y, Rajendran K, Nair GB, Diarrheagenic pathogens in polymicrobial infections. Emerg Infect Dis. 2011;17:606–11. 10.3201/eid1704100939 21470448 PMC3377398

[4] Taniuchi M, Sobuz SU, Begum S, Platts-Mills JA, Liu J, Yang Z, Etiology of diarrhea in Bangladeshi infants in the first year of life analyzed using molecular methods. J Infect Dis. 2013;208:1794–802. 10.1093/infdis/jit507 24041797 PMC3814844

[5] Platts-Mills JA, Liu J, Houpt ER. New concepts in diagnostics for infectious diarrhea. Mucosal Immunol. 2013;6:876–85. 10.1038/mi.2013.50 23881355

[6] Pop M, Walker AW, Paulson JN, Lindsay B, Antonio M, Hossain MA, Diarrhea in young children from low-income countries leads to large-scale alterations in intestinal microbiome composition. Genome Biol. 2014;15:R76. 10.1186/gb-2014-15-6-r76 24995464 PMC4072981

[7] Lindsay B, Ochieng JB, Ikumapayi UN, Toure A, Ahmed D, Li S, Quantitative PCR for detection of *Shigella* improves ascertainment of *Shigella* burden in children with moderate-to-severe diarrhea in low-income countries. J Clin Microbiol. 2013;51:1740–6. 10.1128/JCM.02713-12 23536399 PMC3716050

[8] Platts-Mills JA, Gratz J, Mduma E, Svensen E, Amour C, Liu J, Association between stool enteropathogen quantity and disease in Tanzanian children using TaqMan array cards: a nested case-control study. Am J Trop Med Hyg. 2014;90:133–8. 10.4269/ajtmh.13-0439 24189366 PMC3886409

[9] Kotloff KL, Nataro JP, Blackwelder WC, Nasrin D, Farag TH, Panchalingam S, Burden and aetiology of diarrhoeal disease in infants and young children in developing countries (the Global Enteric Multicenter Study, GEMS): a prospective, case-control study. Lancet. 2013;382:209–22. 10.1016/S0140-6736(13)60844-2 23680352

[10] Bhavnani D, Goldstick JE, Cevallos W, Trueba G, Eisenberg JNS. Synergistic effects between rotavirus and coinfecting pathogens on diarrheal disease: evidence from a community-based study in northwestern Ecuador. Am J Epidemiol. 2012;176:387–95. 10.1093/aje/kws220 22842722 PMC3499114

[11] Yun J-H, Yim D-S, Kang J-Y, Kang B-Y, Shin E-A, Chung M-J, Identification of *Lactobacillus ruminus* SPM0211 isolated from healthy Koreans and its antimicrobial activity against some pathogens. Arch Pharm Res. 2005;28:660–6. 10.1007/BF02969355 16042074

[12] Guandalini S. Probiotics for children with diarrhea: an update. J Clin Gastroenterol. 2008;42(Suppl 2):S53–7. 10.1097/MCG.0b013e3181674087 18520336

[13] Srikanth CV, McCormick BA. Interactions of the intestinal epithelium with the pathogen and the indigenous microbiota: a three-way crosswalk. Interdiscip Perspect Infect Dis. 2008;2008:626827. . Epub 2008 Oct 29. 10.1155/2008/626827 PMC2648619 19259328

[14] Levine MM, Kotloff KL, Nataro JP, Muhsen K. The Global Enteric Multicenter Study (GEMS): impetus, rationale, and genesis. Clin Infect Dis. 2012;55(Suppl 4):S215–24. 10.1093/cid/cis761 23169934 PMC3502311

[15] Kotloff KL, Blackwelder WC, Nasrin D, Nataro JP, Farag TH, van Eijk A, The Global Enteric Multicenter Study (GEMS) of diarrheal disease in infants and young children in developing countries: epidemiologic and clinical methods of the case/control study. Clin Infect Dis. 2012;55(Suppl 4):S232–45. 10.1093/cid/cis753 23169936 PMC3502307

[16] Panchalingam S, Antonio M, Hossain A, Mandomando I, Ochieng B, Oundo J, Diagnostic microbiologic methods in the GEMS-1 case/control study. Clin Infect Dis. 2012;55(Suppl 4):S294–302. 10.1093/cid/cis754 23169941 PMC3502308

[17] Vu DT, Sethabutr O, Von Seidlein L, Tran VT, Do GC, Bui TC, Detection of *Shigella* by a PCR assay targeting the *ipa*H gene suggests increased prevalence of shigellosis in Nha Trang, Vietnam. J Clin Microbiol. 2004;42:2031–5. 10.1128/JCM.42.5.2031-2035.2004 15131166 PMC404673

[18] Ghodsi M, Liu B, Pop M. DNACLUST: accurate and efficient clustering of phylogenetic marker genes. BMC Bioinformatics. 2011;12:271 and. 10.1186/1471-2105-12-271 21718538 PMC3213679

[19] Cole JR, Chai B, Farris RJ, Wang Q, Kulam SA, McGarrell DM, The Ribosomal Database Project (RDP-II): sequences and tools for high-throughput rRNA analysis. Nucleic Acids Res. 2005;33:D294–6. 10.1093/nar/gki038 15608200 PMC539992

[20] Rothman KJ. Epidemiology: an introduction. New York: Oxford University Press; 2012.

[21] Knol MJ, VanderWeele TJ. Recommendations for presenting analyses of effect modification and interaction. Int J Epidemiol. 2012;41:514–20. 10.1093/ije/dyr218 22253321 PMC3324457

[22] Andersson T, Alfredsson L, Källberg H, Zdravkovic S, Ahlbom A. Calculating measures of biological interaction. Eur J Epidemiol. 2005;20:575–9. 10.1007/s10654-005-7835-x 16119429

[23] Zou GY. On the estimation of additive interaction by use of the four-by-two table and beyond. Am J Epidemiol. 2008;168:212–24. 10.1093/aje/kwn104 18511428

[24] Hosmer DW, Lemeshow S. Confidence interval estimation of interaction. Epidemiology. 1992;3:452–6 and. 10.1097/00001648-199209000-00012 1391139

[25] Oksanen J, Blanchet FG, Kindt R, Legendre P, Minchin PR, O’Hara RB, Vegan: Community Ecology Package, 2013 [cited 2014 Apr 8]. http://cran.r-project.org/web/packages/vegan/index.html

[26] Lindsay B, Pop M, Antonio M, Walker AW, Mai V, Ahmed D, Survey of culture, goldengate assay, universal biosensor assay, and 16S rRNA gene sequencing as alternative methods of bacterial pathogen detection. J Clin Microbiol. 2013;51:3263–9. 10.1128/JCM.01342-13 23884998 PMC3811648

[27] Sinha A, SenGupta S, Guin S, Dutta S, Ghosh S, Mukherjee P, Culture-independent real-time PCR reveals extensive polymicrobial infections in hospitalized diarrhoea cases in Kolkata, India. Clin Microbiol Infect. 2013;19:173–80. 10.1111/j.1469-0691.2011.03746.x 22268636

[28] Kwambana BA, Ikumapayi UN, Sallah N, Dione M, Jarju S, Panchalingham S, High genotypic diversity among rotavirus strains infecting Gambian children. Pediatr Infect Dis J. 2014;33(Suppl 1):S69–75. 10.1097/INF.0000000000000087 24343618

[29] Lynn HS, McCulloch CE. When does it pay to break the matches for analysis of a matched-pairs design? Biometrics. 1992;48:397–409. 10.2307/2532299 1637969

[30] Hansson L, Khamis HJ. Matched samples logistic regression in case-control studies with missing values: when to break the matches. Stat Methods Med Res. 2008;17:595–607 . 10.1177/0962280207082348 18375456

