# Supplementary material for: Microbiota That Affect Risk for Shigellosis in Children in Low-Income Countries
Source: Emerg Infect Dis. 2015 Feb;21(2):242–50. doi: 10.3201/eid2101.140795 (PMC4313639; doi:10.3201/eid2101.140795)
Supplement: Supplementary file 1 — Technical Appendix. Supplementary information regarding microbiota tested for their effects on risk for shigellosis in children in low-income countries. [file 14-0795-Techapp-s1.pdf]

DOI article: http://dx.doi.org/10.3201/eid2102.140795

Microbiota that Affect Risk for Shigellosis in Children in Low-Income Countries

Technical Appendix

Supplementary information regarding microbiota tested for their effects on risk for shigellosis in children in low-income countries

Technical Appendix Table 1. Association between pathogens and levels of *Shigella* spp./EIEC *ipaH* gene in children with diarrhea and controls in low-income countries*

Pathogens	High level, no. (%)	Low level, no. (%)	OR (95% CI)	p value
Cases				
Rotavirus	14 (8)	169 (17)	0.31 (0.17–0.55)	1.06×10^{-6}
Norovirus GI or GII	32 (12)	95 (9)	1.41 (0.91–2.18)	0.12
Giardia lamblia	43 (16)	187 (18)	0.69 (0.47–1.00)	0.05
Cryptosporidium spp.	24 (9)	118 (12)	0.84 (0.53–1.34)	0.46
tEPEC	27 (10)	92 (9)	1.15 (0.71–1.86)	0.58
EAEC	58 (21)	195 (19)	1.37 (0.97–1.93)	0.07
ETEC	32 (12)	149 (15)	0.85 (0.56–1.30)	0.46
Campylobacter jejuni	68 (24)	273 (27)	1.01 (0.74–1.38)	0.94
Controls				
Rotavirus	1 (1)	40 (2)	0.31 (0.04–2.33)	0.26
Norovirus GI or GII	13 (10)	128 (8)	1.34 (0.73–2.45)	0.34
G. lamblia	25 (20)	348 (22)	0.81 (0.51–1.29)	0.38
Cryptosporidium spp.	9 (7)	78 (5)	1.52 (0.74–3.11)	0.25
tEPEC	11 (9)	107 (7)	1.13 (0.53–2.39)	0.75
EAEC	27 (21)	320 (20)	1.13 (0.72–1.77)	0.60
ETEC	11 (9)	137 (9)	1.01 (0.53–1.93)	0.97
C. jejuni	21 (16)	243 (15)	1.15 (0.07–1.89)	0.58

*OR, odds ratio; tEPEC, typical enteropathogenic *Escherichia coli* ; EAEC, enteroaggregative *E. coli*; ETEC, enterotoxigenic *E. coli*.

Technical Appendix Table 2. Interaction between levels of *ipaH* gene and *Lactobacillus* and *Veillonella* taxa identified by 16S rRNA gene sequencing and association with moderate-to-severe diarrhea in children in low-income countries*

Organism	RERI (95% CI)	RERI p value	Multiplicative p value
Lactobacillus DJF RP24	**–2.44 (–3.93 to –0.95)**	**0.001**	0.005
Lactobacillus KLDS 1.0718	**–1.93 (–3.56 to –0.29)**	**0.02**	0.01
Lactobacillus TSK G32.2	**–2.69 (–4.55 to –0.84)**	**0.004**	0.02
Lactobacillus salivarius	–1.02 (–3.18 to 1.14)	0.36	0.03
Lactobacillus ruminis	**–1.92 (–3.36 to –0.47)**	**0.009**	0.05
Veillonella R54	–1.73 (–3.48 to 0.03)	0.05	0.06
Lactobacillus KLDS 1.0713	–2.06 (–4.59 to 0.48)	0.11	0.06
Veillonella BP2.87	1.12 (–0.38 to 2.62)	0.14	0.06
Lactobacillus gastricus	–1.46 (–4.07 to 1.16)	0.27	0.08
Lactobacillus fermentum	–0.61 (–2.83 to 1.62)	0.59	0.08
Veillonella BP1.85	1.78 (–0.09 to 3.65)	0.06	0.09
Lactobacillus gasseri	–1.10 (–2.96 to 0.76)	0.24	0.10
Lactobacillus vaginalis	–1.12 (–3.08 to 1.47)	0.40	0.21
Veillonella AA050	0.84 (–0.65 to 2.34)	0.27	0.21
Lactobacillus mucosae	–0.74 (–2.48 to 1.00)	0.40	0.31
Veillonella HB016	11.19 (–8.17 to 30.55)	0.26	0.34
Veillonella dispar	0.76 (–0.80 to 2.32)	0.34	0.42

Organism	RERI (95% CI)	RERI p value	Multiplicative p value
Veillonella ratti	–0.44 (–1.87 to 0.98)	0.54	0.50
Lactobacillus oris	–0.25 (–3.83 to 3.32)	0.89	0.55
Veillonella BP2.30	–0.64 (–4.46 to 3.18)	0.74	0.58
Veillonella MB5.P17	1.15 (–2.31 to 4.63)	0.51	0.65
Veillonella B9	0.35 (–2.79 to 3.50)	0.83	0.69
Veillonella R57	–0.51 (–2.64 to 1.62)	0.64	0.71
Veillonella F12	0.14 (–4.95 to 5.24)	0.96	0.75
Veillonella atypical	–0.08 (–1.93 to 1.77)	0.94	0.78
Veillonella BP2.47	0.06 (–1.07 to 1.82)	0.94	0.79
Veillonella E4	0.33 (–3.10 to 3.77)	0.85	0.80
Veillonella parvula	–0.13 (–2.04 to 1.78)	0.89	0.80
Veillonella ASCG02	–0.65 (–2.10 to 0.80)	0.38	0.89
Lactobacillus crispatus	–0.36 (–3.34 to 2.62)	0.81	0.97
Veillonella C8	1.19 (–2.98 to 5.36)	0.58	0.97

*RERI, relative excess risk caused by the interaction. The multiplicative p value was estimated by using a logistic regression model with an interaction term between high level of *ipaH* and bacterial taxa and represents significance testing for departure for multiplicativity. The RERI p value represents significance testing for departure from additivity. Values in bold are statistically significant.

Technical Appendix Table 3. Interactions between levels of *ipaH* gene and 4 taxa of *Lactobacillus* and risk for moderate-to-severe diarrhea in children in low-income countries*

Characteristic	Low level of *ipaH*		High level of *ipaH*		OR (95% CI)
	No. with MSD/no. without MSD	OR (95% CI)	No. with MSD/no. without MSD	OR (95% CI)	For high level of *ipaH* within strata of *Lactobacillus* spp.
Lactobacillus ruminis					
Negative	609/894	1.00 (Reference)	190/69	4.30 (3.19–5.80)	4.35 (3.23–5.86)
Positive	414/714	0.82 (0.69–0.96)	87/58	2.20 (1.55–3.12)	2.72 (1.90–3.89)
OR (95% CI) for *L. ruminis* within strata of *ipaH* status	NA	0.82 (0.69–0.96)	NA	0.51 (0.33–0.79)	Expected additive = 2.94
Lactobacillus DJF RP24					
Negative	631/944	1.00 (Reference)	201/69	4.58 (3.41–6.15)	4.63 (3.45–6.22)
Positive	392/664	0.85 (0.72–0.99)	76/58	1.99 (1.39–2.85)	2.38 (1.65–3.44)
OR (95% CI) for DJF RP24 within strata of *ipaH* status	NA	0.85 (0.72–0.99)	NA	0.43 (0.29–0.68)	Expected additive = 5.47
Lactobacillus KLDS 1.0718					
Negative	834/1,364	1.00 (Reference)	243/103	4.10 (3.19–5.26)	4.10 (3.19–5.26)
Positive	189/244	1.25 (1.01–1.55)	34/24	2.42 (1.42–4.13)	2.10 (1.18–3.77)
OR (95% CI) for KLDS 1.0718 within strata of *ipaH* status		1.25 (1.01–1.55)		0.62 (0.35–1.12)	Expected additive = 4.35
Lactobacillus TSK G32.2					
Negative	912/1,489	1.00 (Reference)	269/119	3.89 (3.08–4.92)	3.85 (3.05–4.88)
Positive	111/119	1.40 (1.07–1.85)	8/8	1.60 (0.59–4.30)	1.62 (0.54–4.96)
OR (95% CI) for TSK G32.2 within strata of *ipaH* status	NA	1.37 (1.04–1.81)	NA	0.46 (0.16–1.29)	Expected additive = 4.29

*MSD, moderate-to-severs diarrhea; OR, odds ratio; NA, not applicable.

Technical Appendix Table 4. Interactions between 4 taxa of *Lactobacillus* and pathogens and risk for moderate-to-severe diarrhea in children in low-income countries*

Taxa and pathogen	RERI (95% CI)	RERI p value	Multiplicative p value
Lactobacillus ruminis			
Rotavirus	1.78 (–2.48 to 6.05)	0.41	0.12
Norovirus GI or GII	–0.38 (–0.93 to 0.16)	0.17	0.19
Giardia lamblia	0.02 (–0.25 to 0.29)	0.90	0.84
Cryptosporidium spp.	–0.37 (–1.58 to 0.81)	0.53	0.99
tEPEC	0.12 (–0.51 to 0.75)	0.71	0.52
EAEC	–0.10 (–0.40 to 0.20)	0.51	0.40
ETEC	–0.45 (–1.14 to 0.23)	0.19	0.39
Campylobacter jejuni	0.19 (–0.35 to 0.73)	0.49	0.10
Lactobacillus DJF RP241			
Rotavirus	2.74 (–1.84 to 7.32)	0.24	0.04
Norovirus GI or GII	–0.08 (–0.61 to 0.44)	0.76	0.87
G. lamblia	–0.07 (–0.34 to 0.21)	0.64	0.40
Cryptosporidium spp.	–1.02 (–2.30 to 0.26)	0.12	0.27
tEPEC	–0.17 (–0.81 to 0.48)	0.61	0.81
EAEC	0.15 (–0.14 to 0.45)	0.30	0.39
ETEC	–0.21 (–0.86 to 0.45)	0.54	0.90
C. jejuni	0.06 (–0.50 to 0.62)	0.83	0.27
Lactobacillus KLDS 1.0718			
Rotavirus	7.28 (–5.19 to 19.75)	0.25	0.15
Norovirus GI or GII	–0.16 (–0.97 to 0.65)	0.69	0.66
G. lamblia	0.15 (–0.38 to 0.68)	0.59	0.48
Cryptosporidium spp.	0.64 (–1.20 to 2.48)	0.50	0.57
tEPEC	0.15 (–0.91 to 1.20)	0.78	0.85
EAEC	–0.23 (–0.69 to 0.23)	0.32	0.35
ETEC	0.49 (–0.86 to 1.84)	0.48	0.53
C. jejuni	0.26 (–0.55 to 1.08)	0.53	0.60
Lactobacillus TSK G32.2			
Rotavirus	†	†	0.94
Norovirus GI or GII	0.09 (–1.15 to 1.35)	0.88	0.92
G. lamblia	–0.49 (–1.06 to 0.79)	0.09	0.15
Cryptosporidium spp.	0.56 (–1.70 to 2.84)	0.62	0.72
tEPEC	2.08 (–1.21 to 5.38)	0.21	0.08
EAEC	–0.11 (–0.77 to 0.55)	0.74	0.78
ETEC	0.16 (–1.26 to 1.58)	0.82	0.95
C. jejuni	0.13 (–0.99 to 1.27)	0.82	0.99

*RERI, relative excess risk caused by the interaction; tEPEC, typical enteropathogenic *Escherichia coli* ; EAEC, enteroaggregative *E. coli*; ETEC, enterotoxigenic *E. coli*.
†Did not compute because there were no *Lactobillus* TSK G32.2/rotavirus–positive controls.

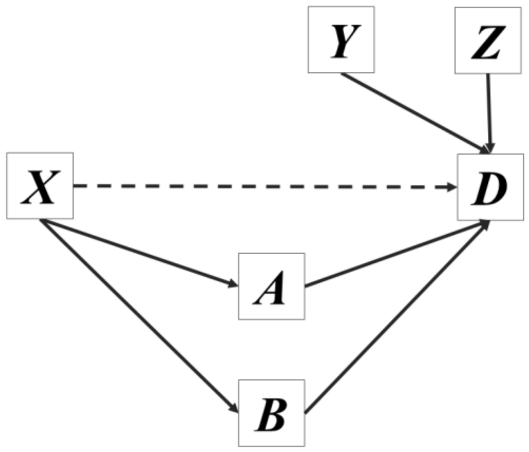

Technical Appendix Figure. Model diagram factors tested for their effects on risk for shigellosis in children in low-income countries. X, high level of *ipaH* gene; D, moderate-to-severe diarrhea; A, age; B, site; Y, co-occurring pathogen; Z, co-occurring microbial taxa.
